# Macrophages and the Tissue Repair Circuit: Homeostasis, Autoimmune Diseases, and Resolution-Based Therapeutic Strategies

**DOI:** 10.3390/cells15151412

**Published:** 2026-08-04

**Authors:** Kenta Mosallanejad, Cedric Hubeau, Annette Schwartz Sterman, Yunhao Tan

**Affiliations:** 1 AbbVie Cambridge Research Center, Cambridge, MA 02139, USA; 2 AbbVie Bioresearch Center, Worcester, MA 01605, USA

**Keywords:** macrophages, fibroblasts, tissue repair, tissue homeostasis, efferocytosis, MerTK, specialized pro-resolving mediators, fibrosis, tissue remodeling, resolution therapy

## Abstract

Tissue repair and regeneration are highly coordinated multicellular processes that rely on the active resolution of inflammation rather than merely its passive cessation. Reciprocal orchestration among stromal, innate, and adaptive immune systems is key to maintaining or restoring tissue homeostasis from pathological perturbations. Macrophages serve as a central nexus of these responses, exhibiting dynamic functional plasticity that extends beyond dichotomous M1/M2 classification. This review explores the evolving, context-dependent roles of macrophages in restoring tissue homeostasis, with a particular focus on efferocytosis and subsequent metabolic rewiring as key drivers of inflammation resolution. Furthermore, we highlight the bi-directional crosstalk between macrophages and heterogeneous fibroblast populations. While these stromal-myeloid interactions are essential for transient matrix remodeling and physiological healing, their sustained activation under inflammatory conditions drives maladaptive repair and fibrotic remodeling. We discuss how the defects and dysregulation of these cellular circuits contribute to the pathogenesis of autoimmune disorders, as exemplified by recent findings in rheumatoid arthritis (RA), systemic sclerosis (SSc), and inflammatory bowel disease (IBD). Finally, we evaluate emerging “resolution therapies” that aim to harness endogenous tissue-reparative programs of macrophages therapeutically to treat autoimmune diseases, including the application of specialized pro-resolving mediators (SPMs) and macrophage reprogramming strategies.

## 1. Introduction

Tissue repair and regeneration are essential for maintaining tissue homeostasis and organismal survival [1,2]. Rather than representing the passive cessation of inflammation, recovery from tissue injury is increasingly recognized as an active, tightly regulated process directed by multicellular resolution programs [3,4]. Among the cellular players that govern this process, macrophages are now recognized as central regulators of both the initiation and resolution of inflammation [5,6,7]. During injury, macrophages not only trigger initial inflammatory responses but also actively support debris clearance, restoration of tissue integrity, and remodeling of the local microenvironment [5]. The outcome of this process is strongly shaped by tissue microenvironments. In normal physiological settings, resolution promotes recovery of tissue function, through regeneration in some organs and adaptive remodeling in others. By contrast, when tissue inflammation persists or resolution signals are inadequate, repair programs can be rewired by chronic inflammation, resulting in tissue remodeling, fibrosis and sustained organ damage [8,9]. Such maladaptive repair programs are increasingly recognized as important components of chronic inflammatory and autoimmune diseases [10,11,12]. In this review, we examine macrophage heterogeneity and function in tissue repair, with particular emphasis on tissue repair driven by dynamic myeloid–stromal interactions. The functional outcome of such interactions depends on cellular state, tissue niche, and timing. We also discuss how these processes become dysregulated in autoimmune disease. Of note, we select rheumatoid arthritis (RA), systemic sclerosis (SSc), and inflammatory bowel disease (IBD) as representative settings in which aberrant macrophage-driven repair manifests as tissue inflammation and tissue remodeling with fibrotic complications. Finally, we highlight emerging therapeutic strategies that aim at enhancing resolution while limiting pathological remodeling.

## 2. Heterogeneity of Macrophages and Their Roles in Tissue Repair: Beyond M1/M2

**Figure 1:**
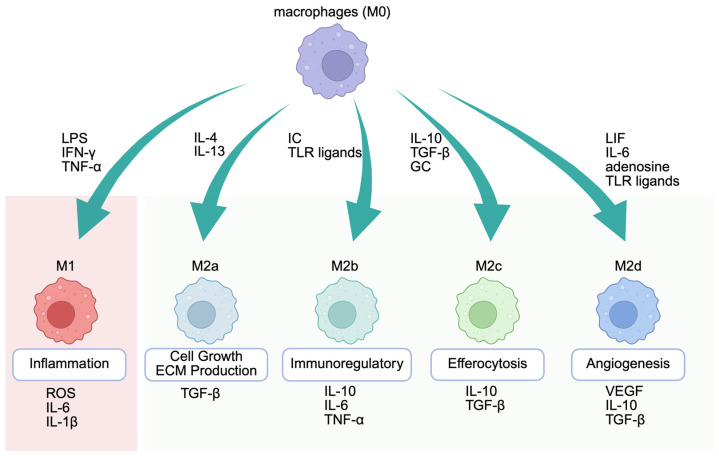
**Historical framework of M1/M2 macrophage polarization.** Macrophages are polarized into M1, M2a, M2b, M2c, and M2d phenotypes upon treatment with the molecules indicated next to each arrow. These distinct macrophage populations exhibit different functions, characterized by their respective cytokine and growth factor production profiles. Selected effector soluble factors are shown at the bottom; this is not an exhaustive list. This figure is included as a historical reference point only; the main text emphasizes that macrophages in tissue repair are more accurately viewed as tissue- and context-dependent states rather than fixed polarization categories.

Macrophages are highly plastic innate immune cells that contribute to host defense, inflammation, tissue homeostasis, and regeneration [5,13]. During tissue injury, macrophage states are heterogeneous and evolve over time, supporting distinct phases, including an early inflammatory phase, subsequent clearance and resolution phase, and finally tissue repair and remodeling phase [5,14,15]. Historically, macrophage functional states have been described using the M1/M2 paradigm [16,17,18]. As shown in Figure 1, distinct molecular cues drive macrophage polarization into M1 and M2 activation states, which are associated with different phenotypic outcomes. In vitro stimulation of macrophages with microbial products such as lipopolysaccharide (LPS), together with interferon gamma (IFN-γ), induces an inflammatory cellular program characterized by robust production of pro-inflammatory cytokines, concomitant with increased expression of major histocompatibility complex (MHC) class II and co-stimulatory molecules CD80 and CD86 [17,19]. In contrast, stimulation with interleukin (IL)-4 and IL-13 induces an alternatively activated M2 program often associated with type 2 immune responses, extracellular matrix (ECM) remodeling, and tissue repair [19]. M2 macrophages have often been further categorized into M2a, M2b, M2c, and M2d [17,19]. M2a macrophages are typically induced by IL-4 and IL-13, which are associated with type 2 inflammation and ECM remodeling. M2b macrophages are induced by immune complexes (IC) in combination with pattern recognition receptor (PRR) agonists [20]. These cells are frequently described as immunoregulatory cells that produce IL-10 while retaining selected pro-inflammatory cytokines. M2c macrophages are induced by IL-10, transforming growth factor (TGF)-β, or glucocorticoids, and are commonly linked to inflammation resolution and tissue repair. M2c macrophages can also produce IL-10 and TGF-β, reinforcing immunoregulatory and reparative programs. These macrophages are marked by increased expression of Mer tyrosine kinase (MerTK), a key efferocytosis receptor, and display enhanced apoptotic cell clearance capacity that contributes to inflammation resolution [21]. The details of MerTK-mediated efferocytosis and subsequent inflammation resolution will be discussed later in this review. M2d macrophages are a more recently identified subset that is distinct from M2a-c, with features resembling tumor-associated macrophages (TAMs). These cells are polarized by tumor microenvironmental cues such as leukemia inhibitory factor (LIF) and IL-6, and upregulate vascular endothelial growth factor (VEGF), TGF-β, and matrix metalloprotease (MMP)-9 to promote angiogenesis and tumor growth [22].

While this framework remains useful as historical shorthand, it does not adequately capture the diversity of tissue macrophages observed in vivo [23]. Indeed, expert consensus has recommended that macrophage activation be described, where possible, according to the source of cells and experimental conditions (e.g., M(IL-4)) rather than by the binary labels alone [19]. Accordingly, in this review, we retain the M1/M2 nomenclature only in a limited historical context and instead emphasize tissue-, niche-, and context-dependent macrophage states.

In keeping with this notion, it is now widely recognized that macrophages in vivo cannot be reduced to a simple binary classification but are highly heterogeneous [24]. They exist as a highly plastic continuum, often demonstrating mixed phenotypes [5,13,16,24,25]. Their roles in tissue repair are shaped by cytokine exposure, developmental origin, niche-derived signals, and the temporal phase of injury and resolution [14,26]. This complexity is especially important when interpreting macrophage programs identified in single-cell datasets, because marker-defined populations may reflect transient or tissue-specific states, rather than fixed lineages.

Tissue repair in vivo is orchestrated by tissue-resident macrophages (TRMs) and infiltrating monocyte-derived macrophages (MDMs) [5,24,27]. TRMs are largely established during embryogenesis from yolk sac or fetal liver precursors and persist through local self-renewal, although in some tissues, such as the intestinal lamina propria, resident populations are continuously replenished by monocyte-derived cells [28,29,30,31]. TRMs are not uniform populations but rather exhibit distinct gene expression profiles and functions across tissues and spaces within a given tissue [24,32,33]. These TRMs maintain tissue homeostasis at steady state by clearing cell debris, regulating metabolism, and releasing trophic factors that support neighboring parenchymal cells [13,14]. In certain tissues, resident macrophages also act as immediate sentinels after microinjury and can physically contain damage and limit inflammatory spread [34]. In the context of more severe injury, however, TRMs sense damage-associated molecular patterns (DAMPs) or pathogen-associated molecular patterns (PAMPs) and release chemokines that recruit neutrophils and monocytes to initiate inflammation [34,35]. Although resident macrophage pools can be markedly reduced after injury, recruited monocytes differentiate locally into MDMs that initially sustain inflammation and later adopt repair-associated functions, including apoptotic cell clearance and ECM remodeling [36,37]. In some settings, these cells can further acquire TRM-like phenotypes and contribute to functional replenishment of the depleted TRM pools. Monocyte engraftment and reprogramming have been proposed to depend largely on tissue niche accessibility, availability, and niche-derived signals that impose tissue-specific functional outcomes [26]. Some of the tissue cues that instruct macrophage identities include NOTCH signaling, GM-CSF, and TGF-β [38,39]. Furthermore, lipids can also contribute to monocyte reprogramming, as evidenced by the recent study demonstrating the lipid-dependent accrual of a distinct MDM subset enriched in cytosolic lipids in the injured murine liver [40]. Additionally, distinct monocyte ontogeny, such as granulocyte-monocyte progenitors (GMPs) or monocyte-dendritic progenitors (MDPs), can gain access to specific tissue niches, thus influencing their phenotypes after they differentiate into TRMs [41]. More recently, deoxyhypusine synthase (DHPS), an enzyme required for the hypusination of eIF5A, has been identified as a cell-intrinsic factor that supports monocyte-to-TRM transition [42]. In short, the list of factors that “train” monocytes to develop tissue specific functions keeps growing.

Recent advances in single-cell RNA sequencing (scRNA-seq) have suggested that tissue repair by infiltrating monocytes does not necessarily require full acquisition of a mature resident macrophage identity. For example, following sterile tracheal epithelial injury, recruited monocyte-derived tracheal macrophages rapidly adopt a pro-regenerative phenotype associated with Arg1 expression, even while retaining immature monocyte-like features [43]. Similarly, in a murine bleomycin-induced lung injury model, a transient macrophage state termed “Mac0” was identified, along the trajectory from recruited monocytes/interstitial macrophages toward alveolar macrophages during repair [44]. This population was characterized by expression of genes including secreted phosphoprotein 1 (Spp1), fibronectin 1 (Fn1), and lipoprotein lipase (Lpl) [44]. Together, these findings suggest that reparative functions can be acquired without full transition to a mature resident macrophage identity, supporting a continuum of monocyte/macrophage states during tissue repair.

An important limitation is that many of these studies rely on mouse models, and therefore it remains to be further explored whether the same or “functional equivalent” monocyte/macrophage subsets exist in humans. Nevertheless, these findings overall support the concept that monocytes and macrophages are highly heterogeneous, and these cell states during post-injury tissue repair are dynamic and transitional, rather than rigidly defined. Consistent with this, the mechanisms by which macrophages contribute to tissue repair are also diverse. Central to many of these functions, however, is the resolution of inflammation through the phagocytic clearance of apoptotic cells. The next section discusses this process, also known as efferocytosis, and how it promotes inflammation resolution and tissue repair.

## 3. Efferocytosis as a Mechanism of Macrophage-Mediated Resolution and Tissue Repair

As aforementioned, macrophages govern the initiation of inflammation and its subsequent resolution. Among the most critical functions of macrophages in this setting is phagocytic clearance of apoptotic cells, a process known as efferocytosis [45,46,47]. In terms of apoptotic cell recognition, the Tyro3-Axl-MerTK (TAM) family of receptor tyrosine kinases is especially important [48]. These TAM receptors recognize growth arrest-specific 6 (Gas6) and Protein S (Pros1), which then bridge phosphatidylserine (PtdSer; the ‘eat-me’ signal) exposed on the outer leaflet of apoptotic cell membranes, promoting receptor oligomerization and efferocytosis. While Gas6 engages all three TAM receptors, Protein S (Pros1) preferentially activates Tyro3 and MerTK [48,49]. In macrophages, MerTK is typically associated with homeostatic or pro-resolving efferocytosis, whereas Axl is more prominently induced under inflammatory conditions [50,51]. Given these properties, MerTK-mediated efferocytosis has been studied extensively in the context of tissue homeostasis and repair and is the primary focus here [46].

Engagement of MerTK with its ligands initiates the engulfment of apoptotic cells while also triggering intracellular signaling that favors pro-resolving macrophage functions. Upon ligand binding, MerTK undergoes autophosphorylation and activates signaling pathways that include PLCɣ2, PI3K-AKT, FAK, and downstream cytoskeletal regulators, thereby promoting actin remodeling for phagocytic cup formation and internalization of apoptotic cargo [45,52,53,54]. After engulfment, the phagosomes fuse with lysosomes to generate phagolysosomes, where the apoptotic cell debris is degraded [47].

**Figure 2:**
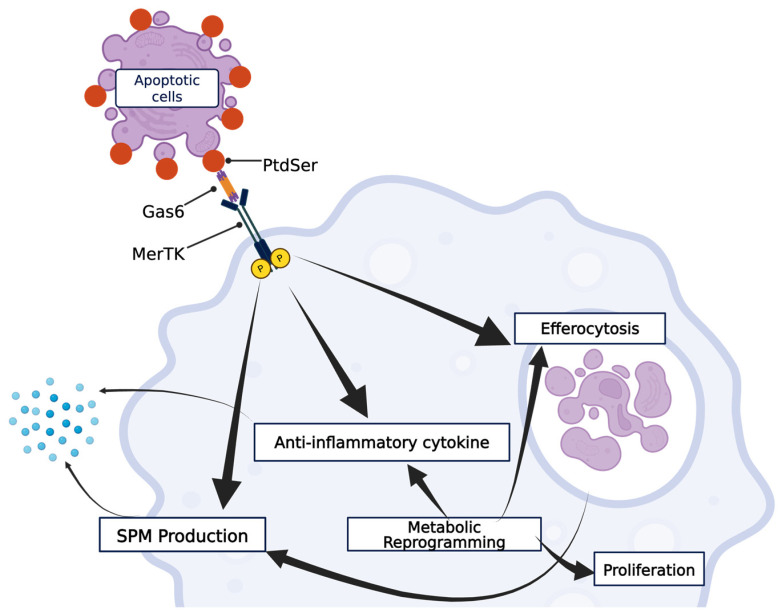
**Pro-resolving effects of MerTK-mediated efferocytosis.** MerTK on the macrophage surface recognizes Gas6 and phosphatidylserine exposed on the apoptotic cell outer leaflet. Upon ligand engagement, MerTK induces efferocytosis via actin remodeling and elicits downstream signaling, driving the production of anti-inflammatory cytokines and specialized pro-resolving mediators (SPMs). Efferocytosis promotes metabolic programs such as fatty acid oxidation (FAO), glycolysis, and amino acid metabolism, which contribute to production of anti-inflammatory cytokines, proliferation of pro-resolving macrophages, and further promoting efferocytosis. The efferocytosis process itself further promotes the release of these mediators.

MerTK signaling and efferocytosis help shift macrophage functional state from inflammatory toward pro-resolving through several mechanisms shown in Figure 2. First, rapid elimination of apoptotic cells prevents secondary necrosis and the release of additional DAMPs [55]. Second, MerTK signaling can directly dampen inflammatory gene expression, including NF-κB-dependent cytokine programs, through a mechanism that depends on kinase activity but appears to be distinct from MerTK autophosphorylation or efferocytosis itself [53]. Third, MerTK activation and efferocytosis redirect macrophage metabolism toward tissue repair. For example, engulfment of apoptotic cells increases intracellular fatty acid availability, stimulating fatty acid oxidation (FAO) and mitochondrial respiration [56]. Increased FAO and electron transport chain elevate intracellular levels of coenzyme nicotinamide adenine dinucleotide (NAD^+^), which activates a SIRTUIN1-dependent signaling cascade that promotes production of the anti-inflammatory cytokine IL-10 through pre-B cell leukemia transcription factor-1 (PBX-1) [56]. Moreover, efferocytosis also promotes a glycolytic program in macrophages. Engulfment of apoptotic cells induces expression of specific solute carrier (SLC) genes, including *SLC2A1* (encoding glucose transporter 1 [GLUT1]) and *SLC16A1* (monocarboxylate transporter 1 [MCT1]), which mediate glucose uptake and lactate export, respectively [57]. Although glycolysis is often associated with pro-inflammatory macrophage activation, efferocytosis-induced glycolysis is mediated by the enzyme 6-phosphofructo-2-kinase/fructose-2,6-bisphosphatase 2 (PFKFB2), distinguishing it from the glycolytic program observed in pro-inflammatory macrophages [58]. PFKFB2-dependent glycolysis increases lactate availability, which in turn elevates expression of efferocytosis receptors such as MerTK, thereby sustaining continuous efferocytosis [58]. Lactate released through SLC16A1 acts in autocrine and paracrine manners to activate AMP-activated protein kinase (AMPK) via the G-protein coupled receptor 132 (GPR132)-protein kinase A (PKA) signaling axis, promoting proliferation of pro-resolving macrophages [57,59]. In addition to glucose and lactate transporters mentioned above, efferocytosis upregulates the SLC36A4 transporter to facilitate tryptophan uptake derived from apoptotic cells, which is metabolized by an indoleamine 2,3-dioxygenase-1 (IDO1)-dependent pathway to promote production of the pro-resolving factors IL-10 and TGF-β, as well as activation of Rac1 that triggers actin polymerization and thereby sustains efferocytosis [60]. Altogether, phagocytic uptake of apoptotic cells by macrophages triggers a wide variety of metabolic programs, including but not limited to FAO, glycolysis, and amino acid metabolisms, which direct pro-resolving cellular processes.

Importantly, changes in macrophage metabolism during efferocytosis induce the biosynthesis of specialized pro-resolving mediators (SPMs), including lipoxins, resolvins, protectins, and maresins [61]. Mechanistically, ligand binding-induced MerTK autophosphorylation in the cytosolic domain activates ERK signaling, which in turn induces SERCA2 expression to lower cytosolic Ca^2+^ and suppress CaMKII-MK2 signaling, promoting retention of 5-LOX in the cytosol. While nucleus-localized 5-LOX favors pro-inflammatory leukotriene production, when retained in the cytosol, 5-LOX promotes SPM production from polyunsaturated fatty acids (PUFAs) such as arachidonic acid (AA), docosahexaenoic acid (DHA) and eicosapentaenoic acid (EPA) [62]. It is also known that apoptotic neutrophils and their microparticles provide AA-, EPA-, and DHA-derived precursors/intermediates to macrophages that engage efferocytosis and stimulate SPM biosynthesis [63]. Furthermore, sterols delivered through efferocytosis can also activate liver X receptor (LXR), a transcription factor that upregulates 12/15-LOX expression to accelerate SPM production [64,65].

Emerging evidence suggests that these SPMs synthesized upon efferocytosis actively resolve inflammatory responses via autocrine or paracrine signals, often through specific G-protein-coupled receptors [61,66]. For example, lipoxin A4 (LXA4) binds to the LXA4 receptor (ALX/FPR2), whereas resolvin D1 (RvD1) signals through ALX/FPR2 and GPR32 [67,68]. They promote pro-resolving leukocyte responses, including enhanced macrophage phagocytosis/efferocytosis and reduced neutrophil recruitment [68,69,70]. Additionally, some DHA-derived SPMs, including D-series resolvins, protectins, and maresins, have been reported to act as biased positive allosteric modulators of the prostaglandin E2 (PGE2) receptor EP4 [71]. This allosteric modulation not only enhances classic Gs-mediated anti-inflammatory signaling but also endows the EP4 receptor with the ability to couple to Gi proteins, converting the cells into phagocytic states. It is noteworthy that SPMs are not the only lipids that mediate tissue repair upon efferocytosis. For instance, in neonatal mice, MerTK-mediated efferocytosis rewires macrophage arachidonic acid metabolism toward thromboxane A2 (TXA2), which promotes proliferation of neighboring cardiomyocytes through the thromboxane-prostanoid (TP) receptor and supports cardiac regeneration [72]. Because TXA2 is an eicosanoid lipid metabolite that is commonly associated with pro-inflammatory and pro-thrombotic roles, it may be better interpreted as a context-dependent pro-regenerative lipid mediator rather than a canonical SPM. Taken together, lipids produced by macrophages upon efferocytosis contribute to inflammation resolution and tissue repair.

In summary, MerTK-mediated efferocytosis promotes resolution not only by removing dying cells, but also by reshaping macrophage signaling, metabolism, and mediator output in ways that can favor tissue repair when appropriately timed and spatially constrained. However, macrophages do not function in isolation; rather, tissue repair is ultimately a multicellular process. Beyond macrophage-intrinsic metabolic rewiring and pro-resolving lipid production, the outcome of repair largely depends on the crosstalk between macrophages and stromal cells. The communication between these two cell types supports transient matrix remodeling and restoration of tissue architecture in physiological settings but can also contribute to aberrant repair when sustained or distorted under chronic inflammation. The next section therefore examines macrophage-fibroblast crosstalk in physiological and aberrant tissue repair.

## 4. Macrophage-Fibroblast Crosstalk That Regulates Tissue Repair

Macrophages and stromal cells, especially fibroblasts, form a functional unit that maintains tissue integrity and facilitates tissue repair responses [73,74,75]. With macrophage biology as the central focus of this review, this section emphasizes macrophage–fibroblast crosstalk in tissue repair, whereas more fibroblast-centered discussions have been reviewed elsewhere [75,76]. Importantly, this reciprocal interaction does not occur just after injury. At steady-state, fibroblasts provide trophic factors such as colony-stimulating factor 1 (CSF1, also known as M-CSF) to direct macrophage survival, transcriptional identity, and population size in the tissue compartment [73,77,78]. Macrophages, in turn, support fibroblast proliferation and metabolism, often through the platelet-derived growth factor (PDGF)-PDGF receptor (PDGFR) axis [73,77]. Broadly, this fibroblast-macrophage reciprocity has been conceptualized as a myeloid-stromal circuit in which fibroblast-derived CSF1 represents a central axis, whereas macrophage-derived trophic inputs that support fibroblast fitness can vary across tissues. In support of this concept, a recent in vivo work in murine skin shows that Dpt-expressing fibroblasts supply CSF1 that sustains local CD64^+^ and CD11c^+^ macrophage populations and that fibroblast-specific ablation of Csf1 alters macrophage composition, as well as perturbs fibroblast metabolism and proliferation [79].

In corroboration of our previous discussion of macrophage functional states, fibroblasts are not a uniform stromal cell type but can be categorized as many heterogeneous populations [75,80]. Cross-tissue single-cell studies in mice and humans have identified fibroblast states that range from relatively conserved populations, including PI16^+^ and COL15A1^+^ fibroblast progenitors, to tissue-specialized and activation-associated derivatives such as LRRC15^+^ myofibroblasts [81,82]. Moreover, structural-cell profiling across organs in mice has shown that fibroblasts harbor organ-specific immune gene programs and epigenetically encoded immune response potential [83]. Thus, fibroblasts are not simply matrix-producing bystanders but actively incorporate and respond to tissue-specific inflammatory and/or reparative cues. Fibroblast heterogeneity is a key determinant of macrophage–fibroblast crosstalk, as the effects of macrophage-derived signals are shaped by both macrophage state and the functional spectrum of the responding fibroblasts [80,81].

The reciprocal activation of macrophages and fibroblasts is crucial to tissue repair after injury. Tissue damage induces inflammatory responses in fibroblasts, which in turn recruit monocytes and macrophages to the injury site through chemokines including CCL2, while macrophages provide trophic mediators that support myofibroblast differentiation and matrix remodeling [84,85]. Activated myofibroblasts, often characterized by alpha-smooth muscle actin (α-SMA) in stress fibers, coordinate wound contraction and provisional ECM deposition that stabilizes damaged tissue and supports restoration of local tissue architecture [86,87].

The crosstalk of macrophages and fibroblasts during tissue repair is highly specific to the subpopulations within each cell type. Upon murine skin injury, CD301b^+^ wound bed macrophages promote the proliferation of adipocyte precursor cells within heterogeneous wound myofibroblast populations, at least in part through secretion of insulin-like growth factor 1 (IGF1) and PDGFC [84]. Consistent with the importance of subset-specific crosstalk, aged mouse skin contains decreased numbers of CD301b^+^ macrophages and adipocyte precursors and consequently exhibits delayed wound healing [84]. This repair circuit is bidirectional, as a recent study leveraging genetic mouse models demonstrated that Dpt^+^ skin fibroblasts supply Csf1 to sustain tissue-reparative CD64^+^ macrophage populations in the skin wound bed [79]. Without this fibroblast-derived signal, the tissue-reparative macrophage pool collapses and is replaced by inflammatory myeloid cells, resulting in defective myofibroblast activation and impaired wound closure [79]. This Csf1-mediated fibroblast-macrophage axis may be relevant in humans, as indicated by the increase in CSF1-expressing fibroblasts and macrophages in systemic sclerosis (SSc) skin samples [79]. In the injured mouse kidney, Arg1^+^ macrophages similarly cooperate with myofibroblasts and endothelial cells during repair to promote local IGF1-dependent epithelial regeneration [88]. In addition to soluble factors, physical cell–cell and cell–matrix interactions play equally essential roles in macrophage-fibroblast communication. An in vitro co-culture study has shown that contractile myofibroblasts generate dynamic deformation fields within the fibrillar collagen matrix, which are detected by macrophage mechanosensing machinery and guide macrophage migration toward fibroblasts, thereby establishing the physical proximity required for efficient crosstalk [89].

Importantly, activation of these repair mechanisms is transient under physiological conditions [5,90]. To prevent excess accumulation of ECM, myofibroblasts undergo deactivation, quiescence, apoptosis, or phenotypic reversion toward the final phase of successful tissue repair [90]. For example, during regression of mouse liver fibrosis induced by carbon tetrachloride (CCl_4_), a substantial fraction of activated myofibroblasts escapes apoptosis and reverts to an inactive state marked by reduced expression of fibrogenic genes [91]. Similarly, during resolution of bleomycin-induced murine lung fibrosis, myofibroblasts transition into lipogenic fibroblasts, or lipofibroblasts, marked by PPARγ expression [92]. Reduced expression of lipofibroblast-associated genes in human idiopathic pulmonary fibrosis (IPF) lung samples indicates the importance of timely phenotypic reversion of myofibroblasts for physiological repair [92]. Macrophages and fibroblasts also cooperate in the resolution phase. For example, during bleomycin-induced mouse lung injury, macrophage-derived tumor necrosis factor-related weak inducer of apoptosis (TWEAK) acts through its receptor fibroblast growth factor-inducible 14 (Fn14) on fibroblasts to protect against fibrosis [93]. TWEAK-Fn14 signaling inhibits TGF-β/Smad-driven fibroblast activation and ECM synthesis while inducing fibroblast-derived chemokines that recruit monocytes/macrophages. These myeloid cells then differentiate into a “pro-regenerative macrophage state” that promotes alveolar epithelial cell proliferation and alveolar regeneration [93]. Elevated Fn14 expression in fibroblasts, together with the presence of a proliferating macrophage population with transcriptional similarity to these pro-regenerative macrophages in human IPF lung samples, suggests that the TWEAK-Fn14 axis may also contribute to resolution of tissue repair machinery in humans, although a causal relationship remains to be determined [93].

Taken together, macrophage–fibroblast crosstalk should be viewed as a conditional circuit—it is beneficial when transient, spatially organized, and properly terminated, but pathogenic when chronic inflammation or niche imbalance rewires the circuit into a hyperactive state. Thus, the distinction between physiological and maladaptive tissue repair lies not in whether macrophages and fibroblasts become activated, but in whether these reciprocal activation states remain self-limited and can be timely terminated. Physiological repair requires actively regulated “OFF” mechanisms of stromal-myeloid activation rather than leaving the repair cellular programs in a perpetual “ON” state. The next section examines how failed termination of this otherwise beneficial tissue repair circuit drives maladaptive tissue remodeling.

## 5. Tissue Remodeling and Fibrosis: Dysregulated Tissue Repair Programs Rewired by Chronic Inflammation

**Figure 3:**
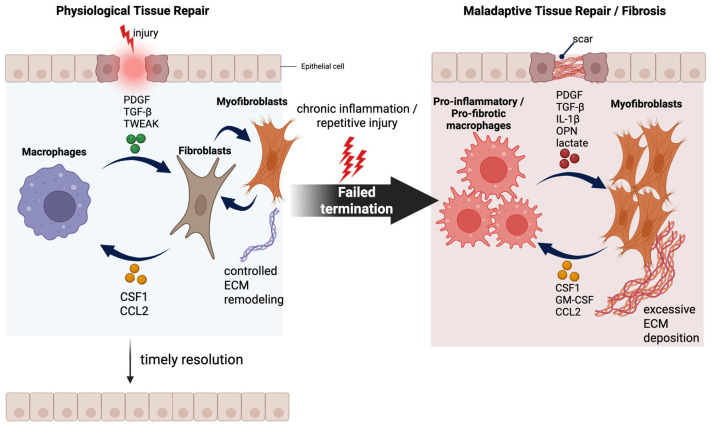
**Macrophage-fibroblast crosstalk in physiological and pathological tissue repair.** In physiological tissue repair, macrophages release growth factors that support fibroblast growth and ECM production (e.g., PDGF, TGF-β). In turn, fibroblasts produce chemoattractants (e.g., CCL2) and provide trophic support (e.g., CSF1) for macrophages. Fibroblast activation, myofibroblast differentiation, macrophage recruitment and provisional ECM deposition are transient and self-limited, thereby stabilizing injured tissue and supporting wound closure. Reciprocal signaling between macrophage and fibroblast subsets coordinates tissue repair. Proper resolution and repair require timely termination of this circuit to deactivate fibroblast and macrophage cellular processes. Under chronic inflammation or repetitive injury, the same stromal–myeloid interactions become sustained or distorted. Inflammatory fibroblasts continue to recruit and instruct myeloid cells, myeloid-derived cytokines lock fibroblasts into pathogenic states, and matrix-remodeling programs become self-reinforcing. In established fibrotic niches, scar-associated macrophage states emerge in close association with activated mesenchymal cells.

During chronic inflammation or repetitive injury, the prolonged activation of macrophages and fibroblasts can lead to an unchecked hyperactivation state that drives scar-forming maladaptive tissue repair, or fibrosis [5]. Activated fibroblasts continue to recruit and instruct monocytes/macrophages, while macrophages continue to reinforce fibroblast activation, matrix remodeling, and profibrotic metabolism [94,95,96]. Figure 3 illustrates how a normally self-limited macrophage–fibroblast repair loop becomes a persistent, pathophysiological remodeling circuit under chronic inflammatory conditions. Importantly, aberrant repair does not reflect the simple presence of activated macrophages and fibroblasts per se, but rather a failure to terminate this normally transient stromal-myeloid repair circuit. In human chronic kidney disease (CKD), an intermediate inflammatory fibroblast population colocalizes with macrophages, and an in vitro study showed that these fibroblasts recruit monocytes and induce the differentiation into FOLR2^+^ macrophages [94]. These macrophages in turn drive the transition of inflammatory fibroblast states toward ECM-secreting myofibroblasts through WNT/β-catenin-dependent signaling [94]. Because detection of this intermediate fibroblast population correlates with human CKD progression, it can be speculated that sustained activation of a normally transitional macrophage–fibroblast repair loop may promote fibrotic niche formation and thereby contribute to disease progression [94]. In heart failure, CCR2^+^ monocytes/macrophages supply IL-1β that acts through fibroblast IL-1 receptor (IL-1R) to stabilize profibrotic fibroblast states through chromatin remodeling involving RELA-MEOX1 and BRD4-dependent programs [95,97]. In hypertrophic scar, macrophage-derived lactate enters fibroblasts through MCT1 and promotes profibrotic histone lactylation [96]. Altogether, under chronic inflammatory conditions, a macrophage-fibroblast repair loop that becomes persistent and self-reinforcing through multiple feed-forward mechanisms can drive pathogenic fibrotic remodeling. Fibrosis is more appropriately viewed as the aberrant persistence of an otherwise self-limited tissue-repair circuit.

Notably, recent advances in single-cell transcriptomics across various organ fibrotic diseases have revealed conserved subpopulations of macrophages within fibrotic niches, often referred to as scar-associated macrophages (SAMs) [98,99,100]. These states were initially defined in cirrhotic liver as TREM2^+^CD9^+^ monocyte-derived macrophages, and related fibrogenic macrophage states across liver and lung were later found to additionally express SPP1, GPNMB, FABP5, and CD63 [98,100]. Their differentiation is promoted by niche-derived signals including type 3 cytokines such as IL-17A and GM-CSF together with TGF-β-associated cues [100]. These macrophages are enriched near activated mesenchymal cells and are associated with deposition of pathogenic ECM, including collagen I, while facilitating remodeling of physiological ECM [98,100]. Several studies have identified macrophages in fibrotic niches that express gene signatures overlapping with SAMs, especially SPP1. For instance, in the context of heart failure and chronic kidney injury, the chemokine CXCL4, derived from platelets and monocytes, expands SPP1^+^ FN1^+^ARG1^+^ macrophages, which trigger fibroblast activation [101]. Altogether, macrophage subsets expressing SPP1 are enriched in the fibrotic niches, resulting in macrophage-fibroblast circuit malfunction. Due to this nature, these macrophages are often associated with fibrotic disorders. However, this interpretation requires caution. It should be emphasized that the mere expression of these marker molecules does not define macrophage-driven fibrotic abnormalities. Rather, they are more appropriately viewed as indicators of injury-associated or matrix-remodeling states, the functional consequences of which depend on the temporal persistence of inflammation and signals derived from the stromal niche. Indeed, macrophages expressing these SAM-associated markers can also participate in physiological tissue repair and resolution of inflammation. For example, TREM2-expressing macrophages are capable of efferocytosis and contribute to physiological tissue repair processes such as wound healing during skin transplantation and cardiac repair upon myocardial infarction [102,103]. Moreover, in a self-limiting murine arthritis model, Spp1^+^ macrophages restrain fibroblast activation through osteopontin (OPN), a protein encoded by *Spp1* gene [104]. Consistent with this distinction, Spp1^+^Gpnmb^+^ macrophages are detected in both fibrotic and self-resolving murine lung injury models, as well as in human lung diseases [105]. These macrophages contract over time in self-limited repair, whereas they persist in fibrotic settings [105]. Collectively, these findings indicate persistent macrophage activation or aberrant subpopulation expansion, rather than the simple emergence of a specific macrophage state, as a central determinant of fibrotic remodeling. Similarly, MerTK^+^ macrophages, which play roles in resolution of inflammation through efferocytosis as discussed in the previous section, can contribute to disease pathogenesis when they accumulate in a different context, as discussed in Section 6.1 [106].

Overall, the dynamic temporal and spatial context of cell–cell interactions, compounded by tissue microenvironment cues, collectively dictate the functional outcomes of macrophage-fibroblast circuit. At the same time, the available evidence is not equally strong across all conclusions from available reports. Some findings are supported by in vivo mouse studies, whereas others rely on observational analyses of human tissues. Nevertheless, accumulating evidence supports the concept that failure to terminate tissue repair can drive maladaptive remodeling that contributes to the pathogenesis of diverse autoimmune diseases in humans. The next section highlights selected examples of autoimmune diseases in which this macrophage-fibroblast tissue repair circuit becomes dysregulated.

## 6. Failed Resolution and Maladaptive Repair in Autoimmune Disorders

Whereas macrophage-fibroblast reciprocal interactions are essential for maintaining homeostasis and physiological tissue repair after injury, dysregulation of these processes can shape the pathophysiological manifestation of autoimmune diseases. In this section, we focus on rheumatoid arthritis (RA), systemic sclerosis (SSc), and inflammatory bowel disease (IBD) as representative settings in which tissue-specific macrophage repair programs become maladaptive within synovial, skin/internal organ, and gastrointestinal mucosal tissue niches, respectively. The indications discussed here are not intended to be exhaustive. Instead, they were selected on the basis of substantial unmet medical need and as distinct examples of how chronic inflammation redirects normally reparative macrophage–stromal programs toward persistent inflammation and fibrosis. In keeping with the scope of this review, our discussion centers primarily on macrophages, while recognizing that fibroblast heterogeneity is a critical determinant of these interactions and their pathological consequences. A detailed discussion of fibroblast heterogeneity in these autoimmune diseases is beyond the scope of this review and has been reviewed elsewhere [107,108,109].

### 6.1. Rheumatoid Arthritis

Rheumatoid arthritis (RA) is a systemic and chronic autoimmune disease characterized by rheumatoid factor and anti-citrullinated protein antibodies [110]. These autoimmune responses are accompanied by immune cell infiltration into the synovium, resulting in joint inflammation and synovial hyperplasia. Chronic tissue inflammation and remodeling in the synovium lead to the formation of an abnormal hyperplastic synovial tissue known as pannus. Although healthy synovium maintains homeostasis by producing lubricants that protect cartilage as well as providing nutrients to cartilage, in RA, both activated macrophages and fibroblast-like synoviocytes (FLS) produce inflammatory cytokines and matrix metalloproteases (MMPs) that damage cartilage, leading to bone destruction [110].

Consistent with our previous discussion, macrophages in the synovial joint are also heterogeneous, and not all macrophage states in the joint are pro-inflammatory. For example, an epithelial-like CX_3_CR1^+^ tissue-resident macrophage population within the lining layer forms a tight-junction-mediated barrier that restricts inflammation [111]. Additionally, gene signatures of MerTK^+^CD206^+^ TRM subsets, especially the TREM2^high^ and FOLR2/LYVE1^+^ subpopulations, are enriched in negative regulators of inflammation, thereby contributing to joint homeostasis and associating with treatment-induced remission in RA [112].

Notably, these marker-defined macrophage populations display phenotypic plasticity. It is reported that, prior to clinical disease onset, overlapping CD206^+^CD163^+^ and MerTK^+^CD206^+^ macrophage populations acquire CD40 expression and inflammatory features, shifting from a reparative profile toward an activated transitional state [113]. The inflammatory macrophages initiate a pathogenic myeloid-stromal circuit in the RA synovium. Notably, these macrophages express both sets of genes historically called ‘M1’ and ‘M2’ markers, supporting the notion that the macrophage states in vivo exist beyond this binary framework [113]. These inflammatory macrophages secrete epidermal growth factor receptor (EGFR) ligands (e.g., HBEGF) that promote highly invasive, matrix-degrading fibroblast phenotypes. In turn, these fibroblasts secrete prostaglandin E2 (PGE2) that sustains the HBEGF^+^ macrophage population [114]. Additionally, a more recent spatial transcriptomic study has shown that RA synovium remodeling is associated with a phenotypic shift and imbalance between tissue-reparative and inflammatory macrophage states. For instance, the LYVE1^+^CD206^+^ TRM subsets, which form a network with stromal cells in homeostatic joints, decrease in active RA but are restored in patients responding to disease-modifying antirheumatic drugs (DMARDs) [115].

Furthermore, dependent on the tissue cue received, even macrophages expressing tissue-reparative markers can unintentionally partake in the tissue-damaging process. A recent study has discovered that in the RA microenvironment, SELENOP^hi^MerTK^+^CD206^+^ macrophages, enriched in tissue repair gene expression, undergo metabolic stress and lytic cell death called PANoptosis via C1q autocrine signaling [106]. Failing to resolve inflammation, the rupture of these macrophages releases inflammatory mediators and DAMPs, which act as a feed-forward mechanism that sustains inflammation [106]. These findings reinforce the notion that the functional outcomes of macrophages are shaped by the collective cues from the tissue microenvironment, and that the repair process can be rewired by persistent tissue inflammation or tissue damage signals, thereby contributing to pathophysiology.

A complementary spatial transcriptomic study further suggests that maladaptive remodeling in RA can also arise from dysregulated activation of myeloid-stromal interactions. SPP1^hi^ macrophages accumulate in fibrin-rich lining regions adjacent to proliferating fibroblasts in RA patient synovium [116]. This observation is consistent with prior cell atlas-scale deconstruction of RA synovium showing the enrichment of SPP1^+^ macrophages in a myeloid-dominant inflammatory subtype [117]. Although the gene expression profile of these cells overlaps substantially with profibrotic SAM markers in the liver and lung, their surrounding extracellular matrix is largely devoid of dense, highly ordered collagen [116]. These SPP1^hi^ macrophages participate in fibrin polymer clearance through fibrinolytic and phagocytic activities. In addition, they promote FLS proliferation, thereby contributing to pannus hyperplasia [116]. Altogether, these findings support the notion that expression of SAM-associated markers does not automatically define macrophages as profibrotic. Furthermore, they reinforce the concept that sustained activation of myeloid-stromal circuit can drive maladaptive tissue remodeling.

### 6.2. Systemic Sclerosis

Systemic sclerosis (SSc), also known as scleroderma, is an autoimmune disease characterized by vascular damage, autoantibodies against nuclear antigens such as RNA polymerase and topoisomerase, dysregulated immune responses, and fibrosis of skin and internal organs such as the lung [118]. Based on the extent of their skin involvement, patients are classified as either limited cutaneous SSc (lcSSc) or diffuse cutaneous SSc (dcSSc) subsets [119]. In SSc, monocytes and macrophages contribute to inflammation upon activation by type I interferon (IFN) and other inflammatory cues and further promote fibrosis [120,121,122,123,124]. Indeed, macrophages in SSc tissues, including lungs in SSc-associated interstitial lung disease (SSc-ILD), undergo phenotypic conversion and express mixed patterns of both historical M1 and M2 markers, reinforcing the view that the M1/M2 binary classification is insufficient to fully characterize macrophage populations in human disease settings [125].

Similar to other autoimmune disorders, SSc shows dysregulated tissue-reparative functions of macrophages. Efferocytosis, the key pro-resolving process of macrophages as discussed above, is attenuated in SSc macrophages [126]. In agreement with this, in vitro polarization of macrophages with CXCL4, a hallmark chemokine in SSc that is secreted by platelets and plasmacytoid dendritic cells (pDCs), downregulates phagocytic receptor expression and thereby inhibits efferocytosis [127]. Meanwhile, the macrophage-fibroblast repair circuits appear to be sustained and overactivated in SSc, leading to persistent ECM deposition and myofibroblast activation [128,129,130]. A recent study further supports this concept by showing that SSc skin fibroblasts express elevated levels of CSF1, which promotes macrophage survival and correlates with increased macrophage abundance and disease severity, although a clear causal relationship remains to be established [79]. Furthermore, *SPP1*, the gene often associated with profibrotic activation status of macrophages, is upregulated in SSc macrophages [128,129,131]. It is noteworthy that CXCL4 induces SPP1^+^ macrophages in the context of renal failures and myocardial infarction, raising the possibility that it may also induce SPP1^+^ profibrotic macrophages in SSc pathogenesis.

Recent spatial studies have suggested that this macrophage-fibroblast crosstalk is not only present in late fibrotic SSc but also prominent in early disease. Multimodal analyses of early, untreated SSc skin have identified a proinflammatory vascular niche featuring macrophage-fibroblast co-localization [132]. Within this niche, monocyte-derived MRC1^+^ macrophages expressing PDGF are in proximity with PDGFR^hi^Thy1^hi^ fibroblasts around the vasculature [132]. These macrophage subclusters in early SSc skin are characterized by IL-6-JAK-STAT signaling and oxidative phosphorylation (OXPHOS)-associated metabolic programs. In addition, macrophage-derived TGF-β and PDGF activate SSc-dominant fibroblast subclusters [132]. Complementing these findings, another spatial omics study has identified expansion of fibrotic niches in SSc skin, with myofibroblast and macrophage enrichment [133]. Within the expanded niche, activated fibroblasts recruit proinflammatory macrophages through CXCL12/CXCR4 interaction [133]. Altogether, the reciprocal stromal–myeloid signaling circuit is likely one of the key pathogenic axes in SSc, which appears in the early phase of vascular inflammation, and eventually contributes to tissue fibrosis.

### 6.3. Inflammatory Bowel Disease

Inflammatory bowel disease (IBD), comprising ulcerative colitis and Crohn’s disease, is a chronic inflammatory disease of the gastrointestinal tract, in which tissue-infiltrated monocyte-derived macrophages are prominent [134]. Upon injury, pro-resolving macrophages engage in multifaceted processes, such as mitigating inflammation and directly supporting intestinal epithelial cell regeneration through production of trophic and repair factors including IL-10, annexin A1, and extracellular vesicle-packaged WNTs [135]. Furthermore, pro-resolving factors released by macrophages after efferocytosis, including TGF-β, IGF-I, and VEGF, can enhance colon fibroblast migration, proliferation, and contraction, thus promoting fibroblast-mediated tissue repair [136]. In this regard, defective macrophage resolution can compromise stromal repair rather than merely prolonging inflammation [137].

In chronic IBD lesions, the macrophage-fibroblast circuit becomes overactivated. A single-cell spatial analysis has revealed a communication network involving macrophages and inflammatory fibroblasts in UC and CD [138]. The “Inflammation-Dependent Alternative (IDA)” macrophages identified by the single-cell analysis express EGFR ligands such as amphiregulin (AREG), HBEGF, and neuregulin 1 (NRG1). They further co-localize with inflammatory fibroblasts expressing *CSF2* and *CSF3* genes, which encode GM-CSF and G-CSF, respectively [138]. These IDA macrophages are distinct from the historical ‘M1/M2’ classification, consistent with the notion that the simplified M1/M2 dichotomy does not accurately capture macrophage states in human diseases, including IBD [123]. A similar analysis of ileal Crohn’s disease (iCD) has identified a pathogenic cellular module containing IgG plasma cells, inflammatory mononuclear phagocytes, activated T cells, and stromal cells (therefore named GIMAT) associated with resistance to anti-tumor necrosis factor (TNF) therapy. Within this module of iCD, inflammatory macrophages are found in proximity with activated fibroblasts [139]. Notably, these inflammatory macrophages produce oncostatin M (OSM), a member of the IL-6 cytokine family that mediates intestinal fibroblast activation via OSM receptor (OSMR), which has long been correlated with anti-TNF refractory disease state [140].

In stricturing CD, maladaptive repair leads to fibrotic remodeling of the tissue. From a single-cell and spatial transcriptomic perspective, fibrotic intestinal strictures are characterized by increased IgG plasma cells, CCR7^hi^CD4^+^ T cells and inflammation-associated fibroblasts (IAF) [141]. These strictures are enriched with interactions between IL1R1^+^ CCL2^+^ inflammatory fibroblasts and *IL1B*^+^ macrophages, indicating the role of IL-1β in promoting the formation of inflammatory fibroblasts [141]. Moreover, IL1B^+^ macrophages accumulating in CD strictures have been found to secrete AREG to promote proliferation and activation of PI16^+^ fibroblasts expressing high levels of ECM-associated genes [142]. In addition to these studies, recent work has identified that MerTK^+^ macrophages are overactivated by OPN in CD intestinal fibrosis, producing TGF-β that promotes fibroblast activation [143]. Given the pro-resolving roles of MerTK^+^ macrophages described earlier, these findings reinforce the concept that functions of a macrophage subset are not fixed but instead are highly plastic and governed by cues from the tissue microenvironment and adjacent cellular constituents. Unchecked repair responses can drive pathological tissue remodeling.

## 7. Resolution Therapy: Therapeutics Leveraging Tissue Repair Functions of Macrophages

**Figure 4:**
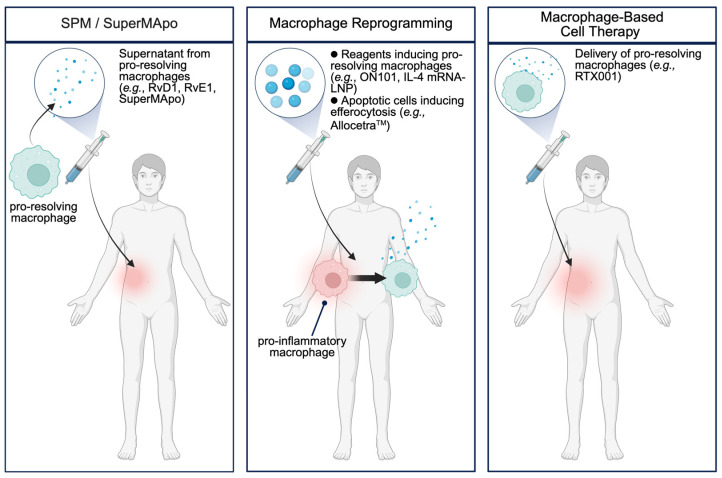
**Advances in therapeutics harnessing pro-resolving functions of macrophages.** Therapeutics associated with pro-resolving macrophages include: (**left**) treatment with specialized pro-resolving mediators (SPMs) or supernatant from pro-resolving macrophages (SuperMApo); (**middle**) reagents and apoptotic cells that induce macrophage transition into a pro-resolving state; (**right**) delivery of pro-resolving macrophages into the body.

The studies discussed above indicate that macrophages are essential for inflammation resolution and subsequent restoration of tissue homeostasis. Therefore, ongoing efforts aim to therapeutically promote these macrophage-mediated resolution processes to restore tissue homeostasis, representing an alternative therapeutic strategy to macrophage depletion or direct blockade of inflammatory mediators. Leveraging the intrinsic pro-resolving cellular programs, these “resolution therapies” encompass a range of strategies, including the use of macrophage-derived soluble mediators such as specialized pro-resolving mediators (SPMs), as well as direct macrophage reprogramming to enhance their pro-resolving functions [144]. The following discussion summarizes recent advances in the development of resolution therapy. The strategies and molecules discussed in this review are summarized in Figure 4 and Table 1, respectively.

The aforementioned SPMs are released from macrophages upon efferocytosis and induce anti-inflammatory responses. Consistently, preclinical studies have shown that SPM treatment attenuates inflammatory responses both in vitro and in vivo. SPMs including RvD1, maresin 1 (MaR1), and protectin DX (PDX), inhibit in vitro inflammatory responses of RA patient-derived FLS as well as alleviate in vivo mouse arthritis [145,146,147]. Indeed, levels of several SPMs have been reported to be reduced in sera from RA patients [145,146,147,166,167,168]. Similarly, RvE1 has been shown to mitigate liver and kidney fibrosis in murine models [148,149,150]. In addition to these and many other preclinical findings, SPMs have also advanced in clinical trials. For example, RX-10001 and RX-10045, RvE1 analogs from Resolvyx Pharmaceuticals, were tested in a phase 1 study in healthy volunteers and in phase 2 studies for dry eye disease and allergic conjunctivitis, respectively [151,152,153]. A benzo-fused ring-modified LXA4 analog BLXA4-ME (Forsyth Institute) was tested in a phase 1/2 trial for gingivitis [154]. Although these SPM analogs were well tolerated and improved some symptoms, they showed mixed efficacy across multiple secondary endpoints, likely due to metabolic instability and limited bioavailability. RTP-026, a synthetic N-terminal Annexin1 peptide, was similarly developed by ResoTher Pharma to stimulate LXA4 receptor FPR2. Preclinical studies showed cardioprotective effects of RTP-026 in acute myocardial ischemia/reperfusion (I/R) mouse models, and the phase 2 study for ST-elevation myocardial infarction (STEMI) is now recruiting [155,156]. Moreover, in addition to these single pro-resolving lipids or peptides, the complex mixture of macrophage-secreted factors is currently developed as a drug. SuperMApo (*super*natant from *m*acrophages eliminating *apo*ptotic cells) is the supernatant derived from macrophages upon efferocytosis, developed by Med’Inn’ Pharma [144]. Preclinical studies have demonstrated pro-healing effects of SuperMApo in murine colitis, arthritis, and tumor models [136,169,170].

Another therapeutic direction is to enhance pro-resolving and pro-regenerative functions of macrophages. For instance, topical application of ON101 cream (Oneness Biotech), consisting of plant-based pharmaceutical ingredients, inhibits pro-inflammatory macrophage activation while promoting tissue-reparative and -regenerative activities [171]. It has been tested in the clinical trials for diabetic foot ulcers (DFUs), in which hyperglycemia disrupts macrophage population balance. An international phase 3 randomized trial demonstrated that ON101 was safe and improved healing outcomes in DFU, and it is currently on the market in some countries [157,158,159]. Macrophage reprogramming may also be achieved by delivering environmental cues. Preclinically, lipid nanoparticles (LNPs) encapsulating IL-4 mRNA promote macrophage polarization and accelerated diabetic wound healing [160]. Moreover, macrophage reprogramming may also be induced by apoptotic cell-based therapies. Allocetra^TM^, which consists of donor-derived mononuclear cells, has been evaluated in primary knee osteoarthritis. Phosphatidylserine (PtdSer) exposed on Allocetra^TM^ provides an “eat-me” signal that promotes efferocytotic uptake by joint macrophages and shifts them towards a homeostatic state. Although the precise mechanisms were not explicitly revealed, such effects may plausibly involve pathways including MerTK-mediated signaling and SPM production discussed in Section 3. Allocetra^TM^ reduced joint pain and showed a favorable safety profile in a phase 1 study and is currently being tested in a phase 2b trial for primary knee osteoarthritis [161,162].

Finally, cell therapies leveraging the pro-regenerative capacity of macrophages are in development. Peripheral infusion of autologous bone marrow-derived macrophages (RTX001; Resolution Therapeutics) was well tolerated and showed encouraging efficacy in a phase 2 clinical trial for cirrhosis in the United Kingdom (MATCH trial) and is advancing into a phase 1/2 trial in the United States (EMERALD trial) [163,164,165]. It is noteworthy that IL-4 mRNA-loaded LNP can be potentially applied to ex vivo generation of pro-reparative macrophages in these cell therapies [172].

## 8. Conclusions

Macrophages have traditionally been viewed as innate immune sentinels that initiate and amplify inflammatory responses upon microbial infection. This review highlights tissue repair as an active, tightly coordinated macrophage-driven program essential for maintaining tissue homeostasis. Of note, macrophages exhibit phenotypic plasticity and molecular features beyond historical M1/M2 classification, as supported by various macrophage activation states observed in human disorders.

Through TAM receptor-mediated clearance of apoptotic cells and the production of SPMs, macrophages actively promote inflammation resolution and tissue repair. Crucially, tissue repair is not driven by macrophages alone; rather, it is coordinated through a bidirectional activation circuit between macrophages and stromal cells, often fibroblasts. Although the myeloid-stromal tissue repair circuit is fundamental both to homeostasis and to responses following tissue injury, excessive activation of this machinery often leads to chronic inflammation and autoimmune diseases. Therefore, it needs to be emphasized that a balanced “ON/OFF” mechanism of tissue repair responses is needed to restore homeostasis. Importantly, a macrophage population bearing markers associated with resolution and repair can nevertheless become ‘tissue-damaging’ when coerced by disease promoting tissue cues. Resolution-based therapeutics aimed at reprogramming macrophages to enhance their pro-resolving pathways and mediators are currently being developed for a wide range of diseases and have shown promising results. Given that persistent inflammation may override pro-resolving macrophage reprogramming in disease settings, the resolution therapies discussed previously may further unleash their potential when used alongside, or following anti-inflammatory treatment. Whether combination or sequential administration is preferable will likely depend on the disease stage, inflammatory burden, and the extent to which the tissue microenvironment remains permissive to resolution. In keeping with this, many autoimmune diseases associated with dysregulated myeloid–stromal tissue repair circuits remain inadequately treated and continue to demand a new generation of transformative therapeutics.

Our understanding of resolution and tissue repair remains limited by several key challenges. For example, many mechanistic insights are derived from mouse injury models, which do not necessarily recapitulate the dynamics of human physiology and diseases. Meanwhile, human single-cell and spatial omics approaches are limited by their cross-sectional nature, providing only snapshots of cellular states at a given time point and therefore lacking temporal resolution. Moreover, inconsistent annotation of cell populations across studies further complicates the interpretation of the mechanisms underlying resolution and tissue repair. In addition, omics-based observations are often correlative, making it difficult to distinguish causal drivers from “bystanders” without functional validation in tractable experimental systems. A key priority for the field will be to pair longitudinal analysis of human tissues with improved cross-study harmonization and experimental models that directly resolve how macrophage–stromal circuit perturbation influences tissue repair, fibrosis, and therapeutic response. Humanized assay platforms such as complex in vitro models (CIVMs), organ-on-chip systems, precision-cut tissue slices, and organoids may provide an important bridge between in vitro systems, animal models, and human disease biology. Integrated with single-cell and spatial omics, these platforms could enable a more causal and temporally resolved dissection of macrophage–stromal circuits across health and disease. A deeper mechanistic understanding of the molecules/markers, pathways, and cell populations associated with disease progression and resolution will therefore be essential for translating increasingly rich omics datasets into actionable targets and transformative therapeutic strategies. Ultimately, realizing the promise of resolution-based therapeutics will require a clearer understanding of the spatiotemporal organization of the macrophage–stromal tissue repair circuits. Future therapies will likely move beyond broadly suppressing inflammation and instead aim to precisely reprogram these circuits to restore the balance between repair and homeostasis while avoiding maladaptive remodeling.

## Figures and Tables

**Table 1:** **Selected therapeutic strategies related to pro-resolving macrophages.** Selected examples of therapeutic approaches discussed in this review aim to promote inflammation resolution and tissue repair by leveraging macrophage pro-resolving programs. These include specialized pro-resolving mediators (SPMs) and related analogs, macrophage-derived secretomes (SuperMApo), macrophage-reprogramming approaches, apoptotic cell-based therapies, and macrophage-based cell therapies. The table is intended to be illustrative rather than exhaustive and highlights representative products, proposed mechanisms, disease settings, and stages of development.

Product	Company	Disease Indication/Model	Therapeutic Modality/Class	Clinical Stage	Reference
RvD1, MaR1, PDX	NA	In vitro human FLS (RvD1) In vivo mouse arthritis (RvD1, MaR1, PDX)	Specialized pro-resolving mediator (SPM)	Preclinical	[145,146,147]
RvE1	NA	In vivo mouse liver and kidney fibrosis	SPM	Preclinical	[148,149,150]
RX-10001	Resolvyx Pharmaceuticals	Dry eye disease	RvE1 analog	Phase 1 (completed)	[151]
RX-10045	Resolvyx Pharmaceuticals	Dry eye disease, Allergic conjunctivitis	RvE1 analog	Phase 2 (completed)	[152,153]
BLXA4-ME	Forsyth Institute	Gingivitis	Lipoxin A4 (LXA4) analog	Phase 1/2	[154]
RTP-026	ResoTher Pharma	ST-elevation myocardial infarction (STEMI)	Annexin A1–based peptide therapeutic	Phase 2 (recruiting)	[155,156]
SuperMApo	Med’Inn’ Pharma	Colitis; arthritis; tumor models	Macrophage-derived secretome	Preclinical	[144]
ON101 cream	Oneness Biotech	Diabetic foot ulcers (DFUs)	Plant-derived macrophage reprogramming agent	Phase 3 (completed); on the market in some countries	[157,158,159]
IL-4 mRNA-loaded LNP	NA	Diabetic wound healing	mRNA delivery	Preclinical	[160]
Allocetra™	Enlivex	Primary knee osteoarthritis	Apoptotic cell-based therapy	Phase 2b (recruiting)	[161,162]
RTX001	Resolution Therapeutics	Cirrhosis	Macrophage cell therapy	Phase 2 (UK; completed) Phase 1/2 (US; recruiting)	[163,164,165]

## Data Availability

No new data were created or analyzed in this study. Data sharing is not applicable to this article.

## Abbreviations

The following abbreviations are used in this manuscript:

AP	adipocyte precursor
AREG	amphiregulin
Arg1	arginase 1
BRD4	bromodomain-containing protein 4
C1q	complement component 1q
CaMKII	calcium/calmodulin-dependent protein kinase II
CCL	C-C motif chemokine ligand
CD	cluster of differentiation
CD	Crohn’s disease
CSF	colony-stimulating factor
DAMPs	damage-associated molecular patterns
dcSSc	diffuse cutaneous systemic sclerosis
DHA	docosahexaenoic acid
DHPS	deoxyhypusine synthase
DMARDs	disease-modifying antirheumatic drugs
ECM	extracellular matrix
EGFR	epidermal growth factor
eIF5A	eukaryotic translation initiation factor 5A
EPA	eicosapentanoic acid
ERK	extracellular signal-regulated kinase
FAK	focal adhesion kinase
FAO	fatty acid oxidation
FAP	fibroblast activation protein
FABP5	fatty acid-binding protein 5
FLS	fibroblast-like synoviocytes
Fn1	fibronectin 1
Fn14	fibroblast growth factor-inducible 14
FOLR2	folate receptor beta
Gas6	growth arrest-specific 6
G-CSF	granulocyte colony-stimulating factor
GIMAT	IgG plasma cells, inflammatory mononuclear phagocytes, activated T cells, and stromal cells
GLUT1	glucose transporter 1
GM-CSF	granulocyte-macrophage colony-stimulating factor
GMPs	granulocyte-monocyte progenitors
GPNMB	glycoprotein nonmetastatic melanoma protein B
GPR132	G protein-coupled receptor 132
GPR32	G protein-coupled receptor 32
HBEGF	heparin-binding EGF-like growth factor
IAF	inflammation-associated fibroblast
IBD	inflammatory bowel disease
IC	immune complex
iCD	ileal Crohn’s disease
IFN	interferon
IGF-1	insulin-like growth factor 1
IgG	immunoglobulin G
IL	interleukin
lcSSc	limited cutaneous systemic sclerosis
LOX	lipoxygenase
LNP	lipid nanoparticle
LIF	leukemia-induced factor
LPS	lipopolysaccharide
LXR	liver X receptor
LXA4	lipoxin A4
LYVE1	lymphatic vessel endothelial hyaluronan receptor 1
Mac0	transient intermediate macrophage state identified in lung injury
MaR1	maresin 1
MCT1	monocarboxylate transporter 1
MDMs	monocyte-derived macrophages
MDPs	monocyte-dendritic cell progenitors
MEOX1	mesenchyme homeobox 1
MerTK	Mer tyrosine kinase
MHC	major histocompatibility complex
MMPs	matrix metalloproteases
MRC1	mannose receptor C-type 1
M1/M2	classically activated/alternatively activated macrophage framework
NOTCH	neurogenic locus notch homolog protein
NF-κB	nuclear factor kappa-light-chain-enhancer of activated B cells
NRG1	neuregulin 1
OPN	osteopontin
OSM	oncostatin M
OXPHOS	oxidative phosphorylation
PAMPs	pathogen-associated molecular patterns
PANoptosis	inflammatory programmed cell death integrating pyroptosis, apoptosis, and necroptosis
PDGF	platelet-derived growth factor
PDGFR	platelet-derived growth factor receptor
PDX	protectin DX
PGE2	prostaglandin E2
PI16	peptidase inhibitor 16
PI3K	phosphoinositide 3-kinase
PLCγ2	phospholipase C gamma 2
POSTN	periostin
Pros1	protein S
PRR	pattern recognition receptor
PtdSer	phosphatidylserine
PUFA	polyunsaturated fatty acids
RA	rheumatoid arthritis
RvD1	resolvin D1
RvE1	resolvin E1
SAMs	scar-associated macrophages
scRNA-seq	single-cell RNA sequencing
SELENOP	selenoprotein P
SERCA2	sarco/endoplasmic reticulum Ca^2+^-ATPase 2
SLC	solute carrier
SPMs	specialized pro-resolving mediators
SPP1	secreted phosphoprotein 1
SSc	systemic sclerosis
SSc-ILD	systemic sclerosis-associated interstitial lung disease
STEMI	ST-elevation myocardial infarction
TAM family	Tyro3-Axl-MerTK receptor family
TAMs	tumor-associated macrophages
TGF-β	transforming growth factor-beta
TNF	tumor necrosis factor
TP	thromboxane-prostanoid receptor
TREM2	triggering receptor expressed on myeloid cells 2
TRMs	tissue-resident macrophages
TWEAK	TNF-like weak inducer of apoptosis
UC	ulcerative colitis
VEGF	vascular endothelial growth factor
WNT	wingless/integrated signaling pathway
